# Results of a patient survey exploring skin symptoms in a menopause clinic

**DOI:** 10.1177/20533691251332403

**Published:** 2025-04-02

**Authors:** Hamisha Salih, Zoe Schaedel, Olivia Hum, Claudia DeGiovanni

**Affiliations:** 1 Dermatology, 8721 University Hospitals Sussex NHS Foundation Trust, Brighton, UK; 2 Brighton and Hove Community Menopause Clinic, Brighton, UK; 3 Foundry Healthcare Lewes, Lewes, UK

**Keywords:** Menopause, HRT, estrogen, peri-menopause, post-menopause

## Abstract

**Background:**

The menopause leads to a reduction in circulating estrogen and progesterone levels, which trigger physiological changes in women. This includes cutaneous changes where these hormonal receptors are present and potential deterioration of previously diagnosed dermatoses. We suspect that the prevalence of skin, hair, vulval and oral symptoms experienced during the menopausal time may be underestimated and may not be considered associated with the menopause in presenting patients.

**Methods:**

A survey was conducted to investigate the dermatological symptoms experienced by women attending a menopause clinic. The Dermatology Life Quality Index (DLQI) was also recorded for each patient.

**Results:**

A high prevalence of skin, hair, vulval and oral symptoms was reported by participants, and these symptoms appear to have an impact on quality of life.

**Conclusion:**

These findings highlight the need for research into the management of these symptoms, including the role of hormone replacement therapy amongst other treatment modalities.

## Introduction

The menopause is defined as the permanent cessation of menstruation, preceded by a peri-menopausal period consisting of several years of irregular menstrual cycles.^1,2^ It is a natural part of physiological ageing driven by declining ovarian function and reduction in levels of estrogen and progesterone hormones. This hypo-estrogenic state leads to several biological changes and persists for the remainder of women’s lives. The effects of this on cardiovascular health and bone density are well-known, as is the role of hormone replacement therapy (HRT) in improving bone density and managing vasomotor symptoms, such as hot flushes. However, the impact of falling estrogen levels on the skin, hair, vulva and mouth are less researched despite the presence of hormonal receptors throughout these sites.^3–7^

Estrogen receptors are present in the skin and impairment of the skin barrier has been noted in estrogen-deficient skin.^8^ Estrogen also impacts collagen levels and the inflammatory state of the skin.^5^ Therefore, it is expected that decreasing estrogen levels may affect normal cutaneous function and may have an impact on common dermatoses. Hypoestrogenism also triggers physiological changes in the mucosal areas, particularly in oral and vulval tissues. Additionally, although the impact of hormone fluctuation on hair is not fully understood, it is thought that the reduction in estrogen levels results in a decrease of anagen hairs.^9^ The changes associated with pregnancy indicate the importance of estrogen on hair growth, as increased growth is observed during pregnancy and hair loss is observed post-partum when estrogen levels fall.^10^ Anti-androgens help in some hair conditions, which support the role of hormone therapy in the management of hair symptoms.^11^

We suspect that skin, hair, vulval and oral symptoms experienced during the menopausal time may be underreported and, when they are reported, may not be considered associated with the menopause in presenting patients. We conducted an online survey with the aim of identifying the symptoms women experience during this peri-menopausal and post-menopausal time, and determine their prevalence in a patient population attending a menopause clinic.

## Method

Patients attending a private specialist menopause clinic were invited to participate in this survey. Patients were seen in the clinic following either self-referral or following referral from their primary care doctor. Following review, they were invited to complete an electronic questionnaire enquiring about skin, hair, genital and oral symptoms. Participation in the questionnaire was voluntary, and ethical approval was not required as this was a quality and service improvement project.

## Results

50 responses were collected in total. 48% of respondents still had menstrual periods, and 7% had a previous hysterectomy. 77% were on hormonal treatments, of which estrogen therapy was most taken.

In terms of skin symptoms, 100% of patients reported at least one skin symptom. The most frequent skin symptoms included itchiness (78%) and dry skin (76%). 58% of participants reported at least one oral symptom, with dry mouth being the commonest reported (36%). 82% of women experienced at least one hair symptom since the onset of their menopause. The most prevalent hair symptoms were hair thinning (54%) and hair shedding (44%). 84% reported experiencing at least one vulval symptom, with the most common being dryness (58%), itchiness (54%) and soreness (38%).

In terms of known dermatological issues, 46% noticed worsening of a previously diagnosed dermatological disorder. In total, 48% of patients reported skin symptoms that they had attempted to manage themselves without consulting their doctor. Open comment boxes demonstrated a common theme amongst participants whereby several over-the-counter topical treatments were trialled before the menopause was identified as the trigger for their symptoms.

*‘Tried for about 18 months with various creams before realising it may be peri-menopause, no-one tells us!’* (Participant quote)

The Dermatology Life Quality Index (DLQI) was also recorded for each patient. The mean DLQI score amongst participants was 5 out of 30 and ranged from 0 to 17 out of 30, indicating that skin symptoms have a varied impact on the quality of life of peri-menopausal and post-menopausal women.

## Discussion

The results of this survey highlight the pervasive effects of the menopause and emphasise the effect the menopause can have on quality of life. Patients appear reluctant to seek medical support for skin symptoms related to the menopause and therefore consequently may not receive optimal treatment.

It is paramount that general practitioners (GPs), dermatologists and menopause specialists are aware of the immense impact the menopause has on the skin, hair, vulva and mouth to improve patient care and the quality of life for affected patients. This is particularly important given that the hypo-estrogenic state driving these symptoms persists for the remainder of women’s lives following the menopause, highlighting the potential longevity of symptoms. 100% of our respondents suffered at least one skin symptom and the impact on their quality of life averaged at 5 out of 30 (mild impact). 48% of the women self-managed their condition without medical guidance, with many not realising that their symptoms could be related to the menopause. This suggests that it would be helpful for GPs and menopause specialists to enquire about skin symptoms when assessing women for peri-menopausal symptoms to ensure early diagnosis and appropriate treatment.

HRT refers to the replacement of declining estrogen and, in women who have a uterus, progestogen is required for endometrial protection. Testosterone is sometimes used in addition for low libido persisting after HRT treatment. Receptors for these hormones have been found across dermatological sites and association between symptoms and the onset of menopause is evident. Despite this, there is little evidence of the impact of HRT on skin conditions. Further research is urgently needed to assess the impact and safety of HRT on cutaneous conditions to enable clinicians to offer high-quality evidence-based advice for patients with skin concerns.

Our survey has several limitations. It was conducted at a single clinic site, with a small sample of respondents. The patients who were approached were attending a menopause clinic and therefore may not be fully representative of all women transitioning through the menopause. Larger scale observational studies including a wider demographic of patients would be helpful in further determining the prevalence of these symptoms and worsening dermatoses amongst menopausal women. The outcomes of high-power clinical trials exploring therapeutic options would also be highly informative to clinicians and will aid in better targeted therapy for patients.

## Conclusion

This survey has highlighted the impact menopause has on women’s lives and the wide range of dermatological symptoms that can arise. To improve quality of life for peri- and post-menopausal women, further research is required to explore management options including HRT for cutaneous, hair, vulval and oral symptoms associated with the menopause.
